# Prognostic Implications of Septal Hypertrophy in Patients with Heart Failure with Mildly Reduced Ejection Fraction

**DOI:** 10.3390/jcm13020523

**Published:** 2024-01-17

**Authors:** Noah Abel, Tobias Schupp, Mohammad Abumayyaleh, Alexander Schmitt, Marielen Reinhardt, Felix Lau, Mohamed Ayoub, Kambis Mashayekhi, Muharrem Akin, Jonas Rusnak, Ibrahim Akin, Michael Behnes

**Affiliations:** 1 Department of Cardiology, Angiology, Haemostaseology and Medical Intensive Care, University Medical Centre Mannheim, Medical Faculty Mannheim, Heidelberg University, 68167 Mannheim, Germanytobias.schupp@umm.de (T.S.);; 2 Division of Cardiology and Angiology, Heart Center University of Bochum, Georgstraße 11, 32545 Bad Oeynhausen, Germany; 3 Department of Internal Medicine and Cardiology, MediClin Heart Centre Lahr, Hohbergweg 2, 77933 Lahr, Germany; 4 Department of Cardiology, St. Josef-Hospital, Ruhr-Universität Bochum, 44791 Bochum, Germany; 5 Department of Cardiology, Angiology and Pneumology, University Hospital Heidelberg, Im Neuenheimer Feld 672, 69120 Heidelberg, Germany

**Keywords:** heart failure with mildly reduced ejection fraction, HFmrEF, septal hypertrophy, interventricular septum, mortality

## Abstract

Cardiac remodeling is frequently observed in patients with heart failure (HF) and serves as an indicator of disease progression and severity. Septal hypertrophy represents an aspect of remodeling that can be easily assessed via an echocardiographic measurement of the interventricular septal end diastole (IVSd), but it has not been evaluated for its prognostic value, particularly in patients with heart failure with mildly reduced ejection fraction (HFmrEF). We retrospectively included 1881 consecutive patients hospitalized with HFmrEF (i.e., a left ventricular ejection fraction of 41–49% and signs and/or symptoms of HF) at one institution during a study period from 2016 to 2022. Septal hypertrophy, defined as an IVSd > 12 mm, was prevalent in 34% of the HFmrEF patients. Although septal hypertrophy was not associated with all-cause mortality at 30 months (median follow-up) (HR = 1.067; 95% CI: 0.898–1.267; *p* = 0.460), it was associated with an increased risk of hospitalization due to worsening HF at 30 months (HR = 1.303; 95% CI: 1.008–1.685; *p* = 0.044), which was confirmed even after multivariable adjustment (HR = 1.340; 95% CI: 1.002–1.792; *p* = 0.049) and propensity score matching (HR = 1.399; 95% CI: 1.002–1.951; *p* = 0.048). Although septal hypertrophy was not associated with the risk of all-cause mortality in patients with HFmrEF, it was identified as an independent predictor of long-term HF-related rehospitalization.

## 1. Introduction

Heart failure (HF) remains a profound challenge to global health, contributing to significant mortality rates and healthcare expenditures [1,2,3,4]. As a complex clinical syndrome, HF arises from various etiologies and pathophysiological mechanisms, leading to impaired cardiac function and alterations in the pressures and dimensions of the heart walls and cavities [5,6]. Structural cardiac remodeling, characterized by changes in the genome expression, neurohormonal activation, and ventricular structure, such as the wall thickness, is observed in many patients with congestive HF and serves as an indicator of disease progression [7,8,9,10,11]. Here, the interventricular septum (IVS) is of vital importance due to its role in maintaining the ventricular integrity and coordinating efficient cardiac pumping [12,13,14]. Previous studies have shown that septal hypertrophy affects the systolic and diastolic left ventricular and atrial functions [15]. treatment of the underlying cardiovascular disease may prevent the progression of septal hypertrophy and improve long-term outcomes [16,17]. Furthermore, septal hypertrophy has been shown to be a predictor of mortality even in patients with normal indexed left ventricular masses [18].

With the 2021 European Society of Cardiology (ESC) guidelines introducing HF with mildly reduced ejection fraction (HFmrEF), which is defined as a left ventricular ejection fraction (LVEF) of 41–49%, a new patient cohort has been formed [19]. This group has typically been excluded from prior randomized controlled trials [20,21,22,23], resulting in limited data, which complicates risk stratification and clinical decision making for patients with HFmrEF.

Transthoracic echocardiography is a commonly used tool to assess patients with HF. Because of its broad accessibility, fast handling, and cost-effectiveness, it has become the standard in the diagnosis and management of HF patients [19]. With advancing technology, such as tissue Doppler imaging or speckle tracking, it is possible to measure additional parameters and indices that can assist in the precise assessment of the heart function [24,25,26]. In clinical practice, however, the interventricular septal end diastole (IVSd) remains a frequently measured parameter to determine the extent of the left ventricular hypertrophy and underlying heart conditions due to its simplicity. Although the IVSd is regularly used in conjunction with other parameters to estimate the severity of the underlying heart disease [27,28], its prognostic value in predicting clinical outcomes has yet to be evaluated, particularly in patients with HFmrEF.

This study seeks to fill this knowledge gap by investigating the long-term prognostic value of the IVSd for the all-cause mortality and HF-related rehospitalization of patients hospitalized with HFmrEF using a large retrospective dataset.

## 2. Materials and Methods

### 2.1. Study Patients, Design, and Data Collection

For the present study, all consecutive patients hospitalized with HFmrEF at one institution were included from January 2016 to December 2022 [29]. Using the electronic hospital information system, all relevant clinical data related to the index event was documented, such as the baseline characteristics, vital signs upon admission, prior medical history, prior medical treatment, length of index hospital and intensive care unit (ICU) stay, laboratory values, and data derived from all non-invasive or invasive cardiac diagnostics and device therapies, such as echocardiographic data, coronary angiography data, and data derived from prior or newly implanted cardiac devices. Every revisit to the outpatient clinic, echocardiographic assessment, rehospitalization related to HF, and adverse cardiac event was documented until the end of the year 2022. The number of HF-related rehospitalizations during follow-up was additionally documented.

The present study was derived from the “Heart Failure With Mildly Reduced Ejection Fraction Registry” (HARMER), representing a retrospective, single-center, all-comers registry including consecutive patients with HFmrEF hospitalized at the University Medical Centre Mannheim, Germany (clinicaltrials.gov identifier: NCT05603390). The registry was carried out according to the principles of the Declaration of Helsinki and was approved by the Medical Ethics Committee II of the Medical Faculty Mannheim, University of Heidelberg, Germany (ethical approval code: 2022-818).

### 2.2. Inclusion and Exclusion Criteria

All consecutive patients ≥ 18 years of age hospitalized with HFmrEF at one institution were included. All included patients underwent at least one standardized transthoracic echocardiography at index hospitalization in the cardiology department, where the diagnosis of HFmrEF was assessed. The diagnosis of HFmrEF was determined retrospectively according to the “2021 ESC Guidelines for the diagnosis and treatment of acute and chronic heart failure” [19]. Accordingly, all patients with LVEFs of 41–49% and symptoms and/or signs of HF were included. The presence of elevated amino-terminal prohormone of brain natriuretic peptide (NT-proBNP) levels and other evidence of structural heart disease were considered to make the diagnosis more likely but were not mandatory for the diagnosis of HFmrEF. The presence of right ventricular dysfunction was defined as a tricuspid annular plane systolic excursion (TAPSE) < 18 mm. A standardized transthoracic echocardiography was performed by cardiologists during routine clinical care in accordance with the current European guidelines [30,31]. The echocardiographic operators were blinded to the final study analyses. For the present study, patients with insufficient data quality of the index echocardiography to measure the IVSd were excluded. No further exclusion criteria were applied. All echocardiographic examinations and reports were reassessed post hoc by two independent cardiologists blinded to the final data analysis to determine the interrater reliability. In cases of ambiguous findings or documentation, the echocardiographic source data were reassessed in individual cases based on the available Digital Imaging and Communications in Medicine (DICOM) files.

### 2.3. Risk Stratification

For the present study, risk stratification was performed according to the presence or absence of septal hypertrophy. Septal hypertrophy was defined as an IVSd > 12 mm as assessed via transthoracic echocardiography during index hospitalization. The IVSd was measured in the parasternal long-axis view in accordance with current international guidelines [32]. Further risk stratification was performed according to the severity of the left ventricular hypertrophy, and patients with IVSds ≤ 10 mm, 10–12 mm, >12–14 mm, and >14 mm were compared. 

### 2.4. Study Endpoints

The primary endpoint was long-term all-cause mortality. Long-term was defined as the median time of the clinical follow-up in months. The secondary endpoints comprised rehospitalization for worsening HF, the in-hospital all-cause mortality, the all-cause mortality at 12 months, cardiac rehospitalization, acute myocardial infarction (AMI), stroke, coronary revascularization, and major adverse cardiac and cerebrovascular events (MACCEs) during long-term follow-up. The all-cause mortality was documented using the electronic hospital information system and by directly contacting state resident registration offices (‘Bureau of Mortality Statistics’). Identification of patients was verified by place of name, surname, date of birth, and registered living address. HF-related hospitalization was defined as a rehospitalization due to worsening HF requiring intravenous diuretic therapy. HF-related rehospitalizations comprised patients with hospitalizations due to worsening HF as the primary cause or as a result of another cause but associated with worsening HF at the time of admission, or as a result of another cause but complicated by worsening HF during its cause. Cardiac rehospitalization was defined as rehospitalization due to a primary cardiac condition, including worsening HF, AMI, coronary revascularization, and symptomatic atrial or ventricular arrhythmias. MACCEs were defined as the composite of all-cause mortality, coronary revascularization, non-fatal AMI, and non-fatal stroke.

### 2.5. Statistical Methods

Quantitative data is presented as means ± standard errors of the means (SEM), median and interquartile ranges (IQRs), and ranges depending on the distribution of the data. They were compared using the Student’s *t*-test for normally distributed data or the Mann–Whitney *U* test for nonparametric data. Deviations from a Gaussian distribution were tested using the Kolmogorov–Smirnov test. Qualitative data is presented as absolute and relative frequencies and were compared using the chi-square test or the Fisher’s exact test, as appropriate. The association between the IVSd and the other laboratory and echocardiographic parameters was measured with Spearman’s correlation. Interrater realiability was tested using kappa statistics. Kaplan–Meier analyses were performed, stratified by the IVSd, and univariable hazard ratios (HRs) were obtained together with 95% confidence intervals (CIs). The prognostic impact of septal hypertrophy was thereafter investigated within multivariable Cox regression models using the “forward selection” option.

Due to the heterogeneous distribution of the baseline characteristics and comorbidities present within the all-comers registry, propensity score matching was performed to create more balanced subgroups and thereafter re-evaluate the prognostic impact of septal hypertrophy. Propensity score matching was applied for the comparison of patients with IVSds > 12 mm vs. ≤12 mm, including the entire study cohort, and a non-parsimonious multivariable logistic regression model was applied. Propensity scores were created according to the presence of the following independent variables: age; sex; body mass index (BMI); prior congestive HF; prior decompensation; prior myocardial infarction; prior percutaneous coronary intervention; chronic obstructive pulmonary disease; arterial hypertension; diabetes mellitus; hemoglobin; NYHA functional class; ischemic cardiomyopathy; AMI; diastolic dysfunction; the left ventricular end-diastolic diameter; beta blockers; SGLT-2 inhibitors; and ACE inhibitors/angiotensin receptor blockers/angiotensin receptor–neprilysin inhibitors at discharge. Based on the propensity score values counted via logistic regression, for each patient, one patient in the control group with a similar propensity score value was found (accepted difference in propensity score values: <5%). Within the propensity-score-matched subgroup, the Kaplan–Meier method was applied, and univariable HRs were obtained together with 95% CIs.

Results of all statistical tests were considered significant for *p* ≤ 0.05. SPSS (Version 28, IBM, Armonk, New York, NY, USA) was used for statistics.

## 3. Results

### 3.1. Study Population

**Figure A1:**
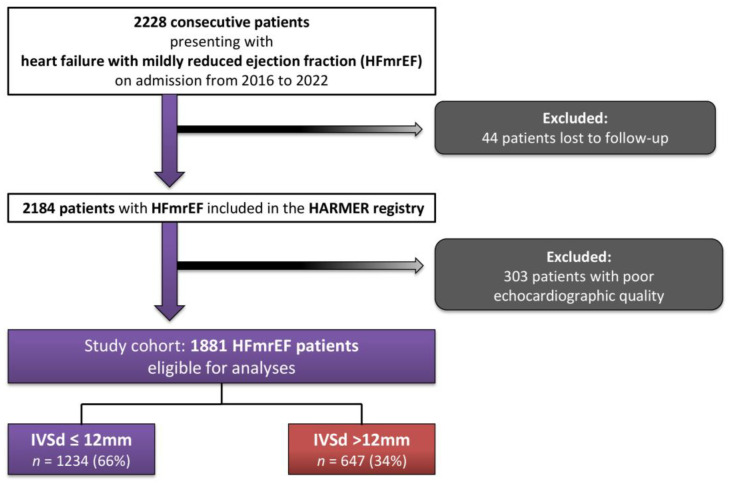
Flow chart of the study population.

From 2016 to 2022, a total of 2228 consecutive patients with HFmrEF were hospitalized at our institution. Totals of 1.97% (*n* = 44) with incomplete follow-up data and 13.6% (*n* = 303) with insufficient data quality to measure the IVSd were excluded (Appendix A Figure A1). The final study cohort comprised 1881 patients hospitalized with HFmrEF with a median IVSd of 12.0 mm (mean: 12.4 mm; IQR: 9.9–14.8 mm). Septal hypertrophy, defined by an IVSd > 12 mm, was present in 34% (*n* = 647) of all patients. The interrater agreement of the IVSd was high (κ = 0.890).

When comparing patients with and without septal hypertrophy, patients with septal hypertrophy were more commonly males (72.3% vs. 62.7%; *p* = 0.001) and presented with higher rates of chronic kidney disease (35.2% vs. 29.2%; *p* = 0.007), arterial hypertension (84.9% vs. 74.8%; *p* = 0.001), and diabetes (42.0% vs. 33.9%; *p* = 0.001) compared to patients without septal hypertrophy (Table 1). In contrast, the rates of pre-existent congestive HF (35.7% vs. 31.8%; *p* = 0.092) and the proportion of patients with HF-related hospitalizations within the last 12 months (11.4% vs. 10.1%; *p* = 0.381) did not significantly differ between the groups. On the contrary, patients with septal hypertrophy had lower rates of chronic obstructive pulmonary disease (8.5% vs. 13.4%; *p* = 0.002). A total of 24.7% of patients with septal hypertrophy suffered from acute decompensated heart failure (vs. 19.8% without septal hypertrophy; *p* = 0.013) and 15.5% suffered from non-ST-segment AMI (NSTEMI) (vs. 11.3% without septal hypertrophy; *p* = 0.010), whereas 9.3% of patients without septal hypertrophy suffered from ST-segment elevation AMI (STEMI) (vs. 6.2% with septal hypertrophy; *p* = 0.019).

As outlined in Table 2, ischemic cardiomyopathy was the most common HF etiology in both groups (60.7% vs. 56.5%), whereas, specifically, the proportion of patients with hypertensive cardiomyopathy (12.7% vs. 6.5%, *p* = 0.001) was higher in patients with septal hypertrophy. In contrast, the rates of primary non-ischemic cardiomyopathies were low (6.0–6.6%). Among the non-ischemic cardiomyopathies, patients with septal hypertrophy had lower rates of dilated cardiomyopathies (1.9% vs. 3.8%; *p* = 0.001), whereas only a minor part of the study population had non-compaction cardiomyopathy (0.2% vs. 0.1%), restrictive cardiomyopathy (0.2% vs. 0.1%), cardiomyopathy related to myocarditis (0.2% vs. 0.3%), takotsubo cardiomyopathy (0.8% vs. 0.8%), myocardial storage disease (0.6% vs. 0.2%), toxic cardiomyopathy (0.2% vs. 0.7%), and constrictive pericarditis (0.2% vs. 0.0%). Finally, 0.5% of patients with septal hypertrophy suffered from hypertrophic obstructive cardiomyopathy, and 2.2% suffered from hypertrophic non-obstructive cardiomyopathy. With regard to the echocardiographic parameters, the left ventricular end-diastolic diameter (LVEDD) was higher in patients without septal hypertrophy (48 mm vs. 49 mm; *p* = 0.001). In line with this, the proportions of patients with aortic stenosis (15.5% vs. 6.5%; *p* = 0.001), regurgitation (5.6% vs. 3.2%; *p* = 0.011), and mitral regurgitation (14.7% vs. 11.5%; *p* = 0.049) were higher in patients with septal hypertrophy. Patients with septal hypertrophy had higher baseline creatinine (1.10 mg/dL vs. 1.05 mg/dL; *p* = 0.001) and amino-terminal pro-brain-type natriuretic peptide (NT-proBNP) levels (3327 pg/mL vs. 2279 pg/mL; *p* = 0.005). Finally, patients with septal hypertrophy were more commonly discharged with angiotensin receptor blockers (26.0% vs. 21.8%; *p* = 0.044), thiazide diuretics (22.0% vs. 15.8%; *p* = 0.001), statins (73.2% vs. 66.8%; *p* = 0.005), and ASA (54.5% vs. 49.4%; *p* = 0.040).

### 3.2. Correlation of IVSd with Clinical and Echocardiographic Parameters

In patients hospitalized with HFmrEF, the IVSd correlated with age (r = 0.102; *p* = 0.001) and body mass index (r = 0.127; *p* = 0.001) (Table 3). Furthermore, the IVSd correlated with the posterior wall thickness (r = 0.683; *p* = 0.001) and aortic jet velocity (r = 0.137; *p* = 0.001), whereas an inverse correlation with the left ventricular end-diastolic diameter (r = −0.094; *p* = 0.001) was observed. With regard to the laboratory data, the IVSd correlated with the creatinine levels (r = 0.107; *p* = 0.001) and hemoglobin (r = 0.048; *p* = 0.039). However, the IVSd showed no correlation with the NT-proBNP levels (r = 0.045; *p* = 0.268) or tricuspid annular plane systolic excursion (r = −0.043; *p* = 0.060).

### 3.3. Prognostic Impact of IVSd in Patients with HFmrEF

**Figure 1:**
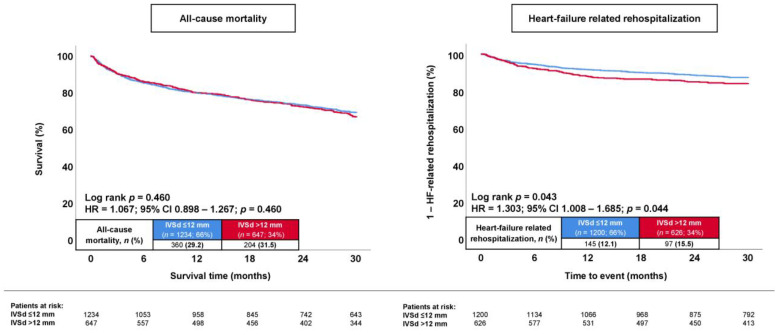
Kaplan–Meier analysis comparing the prognostic impact of septal hypertrophy versus that of no septal hypertrophy on the risk of all-cause mortality (**left panel**) and hospitalization for worsening HF (**right panel**) in patients with HFmrEF.

**Figure 2:**
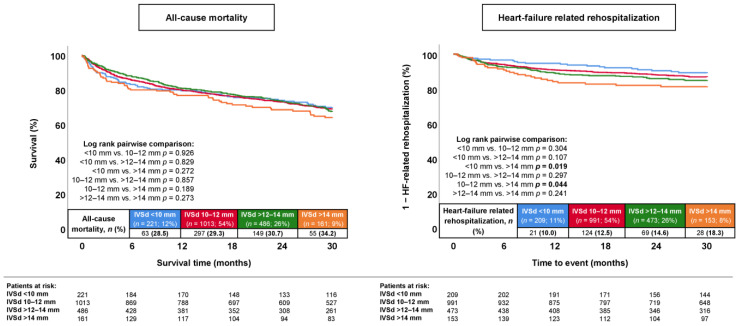
Kaplan–Meier analysis comparing the prognostic impacts of various IVSd categories on the risk of all-cause mortality (**left panel**) and hospitalization for worsening HF (**right panel**) in patients with HFmrEF.

During a median follow-up of 30 months, the primary endpoint, long-term all-cause mortality, occurred in 31.5% of patients with septal hypertrophy and in 29.2% of patients without septal hypertrophy (log rank: *p* = 0.460) (Figure 1; left panel). Accordingly, the presence of septal hypertrophy was not associated with the risk of long-term all-cause mortality (HR = 1.067; 95% CI: 0.898–1.267; *p* = 0.460). In contrast, the long-term risk of HF-related rehospitalization at 30 months was higher in patients with septal hypertrophy (15.5% vs. 12.1%; log rank: *p* = 0.043; HR = 1.303; 95% CI: 1.008–1.685; *p* = 0.044) (Figure 1; right panel). Even when stratified by the severity of the septal hypertrophy, the IVSd was not associated with the risk of long-term all-cause mortality (log rank: *p* ≥ 0.189) (Figure 2, left panel), whereas the risk of HF-related rehospitalization was increased in patients with IVSds > 14 mm compared to <10 mm (18.3% vs. 10.0%; log rank: *p* = 0.019; HR = 1.939; 95% CI: 1.101–3.414; *p* = 0.022) and 10–12 mm (18.3% vs. 12.5%; log rank: *p* = 0.044; HR = 1.519; 95% CI: 1.008–2.289; *p* = 0.046) (Figure 2, right panel).

Regarding the key secondary endpoints, the rates of in-hospital all-cause mortality (3.2% vs. 2.8%; *p* = 0.223), cardiac rehospitalization (23.6% vs. 21.1%; *p* = 0.209), revascularization (6.5% vs. 6.9%; *p* = 0.766), and MACCE at 30 months (39.4% vs. 36.2%; *p* = 0.320) were comparable in patients with and without septal hypertrophy (Appendix A Table A1).

Even after multivariable adjustment for the patients’ characteristics and comorbidities, the presence of septal hypertrophy was still not associated with the risk of long-term all-cause mortality (HR = 1.001; 95% CI: 0.985–1.017; *p* = 0.930) (Table 4). However, septal hypertrophy was associated with a higher long-term risk of rehospitalization for worsening HF compared to patients without septal hypertrophy (HR = 1.340; 95% CI: 1.002–1.792; log rank: *p* = 0.049) (Table 4). Furthermore, prior decompensated HF within 12 months (HR = 1.591; 95% CI: 1.080–2.346; *p* = 0.019) and an NYHA functional class (HR = 1.354; 95% CI: 1.174–1.560; *p* = 0.001) increased the long-term risk of HF-related rehospitalization, whereas higher hemoglobin levels (HR = 0.889; 95% CI: 0.833–0.949; *p* = 0.001) were associated with a lower long-term risk of HF-related rehospitalization. When stratified by the severity of the septal hypertrophy, specifically, an IVSd > 14 mm was associated with a higher long-term risk of rehospitalization due to worsening HF compared to patients without septal hypertrophy (HR = 2.261; 95% CI: 1.153–4.433; *p* = 0.018) (Table 5).

### 3.4. Propensity-Score-Matching Analysis

**Figure 3:**
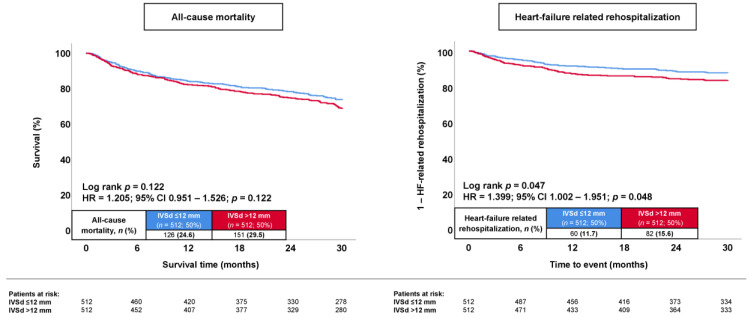
Kaplan–Meier analysis comparing the prognostic impact of septal hypertrophy versus that of no septal hypertrophy on the risk of all-cause mortality (**left panel**) and hospitalization for worsening HF (**right panel**) in patients with HFmrEF after propensity score matching.

In relation to the all-comers study design that included consecutive patients with HFmrEF, an additional propensity-score-matching analysis was performed to further investigate the prognoses of patients with and without septal hypertrophy. After the propensity score matching (*n* = 512 matched pairs with and without septal hypertrophy), patients with septal hypertrophy were still older (77 vs. 75 years; *p* = 0.008) and presented with higher rates of arterial hypertension (86.1% vs. 81.3%; *p* = 0.034) and diabetes mellitus (42.6% vs. 35.9%; *p* = 0.030) (Table 1 and Table 2). The risk of long-term all-cause mortality was still not affected by septal hypertrophy after the propensity score matching (29.5% vs. 24.6%; log rank: *p* = 0.122; HR = 1.205; 95% CI: 0.951–1.526; *p* = 0.122) (Figure 3; left panel). However, the presence of septal hypertrophy was still associated with a higher long-term risk of HF-related rehospitalization (15.6% vs. 11.7%; log rank: *p* = 0.047; HR = 1.399; 95% CI: 1.002–1.951; *p* = 0.048) compared to patients without septal hypertrophy (Figure 3; right panel).

## 4. Discussion

The present study investigates the prevalence and prognostic value of septal hypertrophy on long-term prognosis in consecutive patients hospitalized with HFmrEF using a large retrospective registry-based dataset from 2016 to 2022. Septal hypertrophy, defined by an IVSd > 12 mm, was prevalent in 34% of all patients hospitalized with HFmrEF. Patients with septal hypertrophy were more commonly males and presented with higher rates of arterial hypertension, diabetes, chronic kidney disease, and valvular heart diseases. Although the presence of septal hypertrophy was not associated with the primary endpoint, all-cause mortality at 30 months, septal hypertrophy was associated with an increased risk of HF-related rehospitalization at 30 months, which was demonstrated using multivariable Cox regression analyses and propensity score matching. Specifically, patients with higher IVSds (i.e., >14 mm) were associated with the highest risk of HF-related rehospitalization.

Various pathologies, like chronic volume and pressure overload due to arterial hypertension or aortic stenosis, ischemic diseases, storage diseases, and hypertrophic or dilated cardiomyopathies, are considered to cause myocardial hypertrophy [33,34]. While the etiology may vary, the resulting hypertrophy can temporarily compensate for the underling alterations in some cases. In the long term, however, it may lead to cardiac remodeling, perfusion abnormalities, and diastolic dysfunction, further deteriorating the ventricular function [35].

While it remains unclear whether septal hypertrophy itself can cause HF, our observation of an increased risk of HF-related rehospitalization in HFmrEF patients with septal hypertrophy corresponds to the established concept of cardiac remodeling to impair the systolic function and increase the susceptibility to acute decompensation [36,37,38]. Supporting evidence that shows that septal hypertrophy is not only a bystander of HF but also a contributor to it is provided in a study by Gardin et al. in which the IVSd was an independent predictor of the incident HF (HR = 1.87; 95% CI: 1.13–3.11; *p* < 0.001) in a population aged ≥65 years [39]. While the prognostic value of the IVSd has not been evaluated in detail so far, various methods to measure LV hypertrophy have been used to assess the prognosis of LV remodeling. An analysis of the studies of the left ventricular dysfunction (SOLVD) registry and trials by Quinones et al. used the cube formula applied to the IVSd and posterior wall thickness to estimate the LV mass in patients with LV dysfunction. In contrast to our findings, the LV mass was associated with both all-cause mortality (HR = 1.62; 95% CI: 1.62–4.66; *p* = 0.0002) and cardiovascular hospitalization (HR = 1.81; 95% CI: 1.39–2.36; *p* = 0.0001) [40]. A different approach was used in a post hoc analysis of the CHARM trials, where Hawkins et al. assessed the LV hypertrophy via a 12-lead electrocardiogram (ECG). Using this method, the LV hypertrophy was an independent predictor of cardiovascular death in both patients with HF and LVEFs > 40% (HR = 1.58; 95% CI: 1.05–2.37; *p* = 0.029) and patients with LVEFs ≤ 40% (HR = 1.68; 95% CI: 1.09–2.58; *p* = 0.019), but not for hospitalization related to HF (HR = 1.03, 95% CI: 0.73–1.44, *p* = 0.884 and HR = 1.35, 95% CI: 0.94–1.95, *p* = 0.105, respectively) [41]. Interestingly, the baseline characteristics differed between both groups. Arterial hypertension (81.1% vs. 61.6%) and atrial fibrillation (AF) (32.2% vs. 24.1%) were more common, and myocardial infarction (37.4% vs. 44.0%) and mitral regurgitation (18.9% vs. 25.7%) were less common in the group with LVEFs > 40%. These differences may account for the different clinical characteristics of the HF phenotypes and imply distinct etiologies of septal hypertrophy according to the LVEF, although the outcomes appear to be comparable between the two groups.

In line with this, a subsequent analysis of the PARAGON-HF trial reported comparable echocardiographic characteristics in patients with HF and LVEFs ≥ 45%. High proportions of the patients had comorbid arterial hypertension (94%) and AF (35%), but only 21% had a prior myocardial infarction [42]. In line with the findings of the present study, Shah et al. demonstrated an increased risk of HF hospitalization (HR = 1.35; 95% CI: 1.01–1.81; *p* = 0.04) in patients with increased mean wall thicknesses, while no association with cardiovascular death (HR = 1.48; 95% CI: 0.91–2.40; *p* = 0.11) was found.

In contrast, a post hoc analysis of the Atrial Fibrillation Follow-up Investigation of Rhythm Management (AFFIRM) trial suggested that the IVSd was able to independently predict both the all-cause mortality (HR = 1.46; 95% CI: 1.14–1.86; *p* = 0.003) and the risk of stroke (HR = 1.89; 95% CI: 1.17–3.08; *p* = 0.010) in patients with atrial fibrillation [43]. It is worth mentioning that the AFFIRM trial only included patients with at least a moderate risk of stroke or death according to age and clinical risk factors, possibly limiting the comparability to unselected cohorts.

While our study defines the previously unclear prognostic value of septal hypertrophy in HFmrEF patients, further evidence that septal hypertrophy not only increases the susceptibility to HF hospitalization but also increases the mortality in selected patients is provided by a study conducted by Huang et al. [18]. In a prospective cohort of coronary artery disease (CAD) patients, septal hypertrophy was associated with a higher risk of all-cause mortality (HR = 1.49, 95% CI: 1.00–2.23, *p* = 0.05 for mildly abnormal IVS thickness, and HR = 2.13, 95% CI: 1.29–3.54, *p* = 0.003 for moderately to severely abnormal IVS thickness, compared to patients with normal IVS thickness) [18]. While the increased mortality contrasts with the present study, CAD patients may be more vulnerable to the deleterious effects of septal hypertrophy due to the additive burden of CAD and its associated complications. This underscores the complex interplay between comorbidities, cardiac structural changes, and clinical outcomes, emphasizing the need for tailored management strategies in different patient populations.

## 5. Study Limitations

This study has several limitations. Due to the retrospective and single-center study design, the results may have been influenced by measured and unmeasured confounding. In the absence of clear guideline recommendations for the treatment of patients with HFmrEF, cardiac magnet resonance imaging was performed on a minor part of the study population only and was not taken into account in the present study. Related to the overall low rates of patients with primary non-ischemic cardiomyopathy and hypertrophic cardiomyopathy, no further sub-analyses could be performed according to the prognostic impact of the IVSd in this specific population. HF-related and cardiac rehospitalizations were assessed at our institution only. In relation to the inclusion period until 2022, not all patients could have been followed for 30 months. Therefore, the primary endpoints were assessed in accordance with the median follow-up time of the study population. Finally, causes of death beyond index hospitalization were not available for the present study.

## 6. Conclusions

In summary, septal hypertrophy (i.e., an IVSd > 12 mm) was present in one out of three patients with HFmrEF. While the presence and absence of septal hypertrophy showed comparable risks of long-term all-cause mortality, septal hypertrophy was associated with a higher risk of HF-related rehospitalization at 30 months.

## Figures and Tables

**Table 1:** Baseline characteristics.

	Without Propensity Score Matching					With Propensity Score Matching				
	IVSd ≤ 12 mm (*n* = 1234)		IVSd > 12 mm (*n* = 647)		*p* Value	IVSd ≤ 12 mm (*n* = 512)		IVSd > 12 mm (*n* = 512)		*p* Value
Age, median (IQR)	75	(63–82)	77	(65–84)	**0.002**	75	(63–82)	77	(66–84)	**0.008**
Male sex, n (%)	774	(62.7)	468	(72.3)	**0.001**	352	(68.8)	368	(71.9)	0.274
Body mass index, kg/m^2^, median (IQR)	26	(24–30)	27	(24–31)	**0.002**	27	(24–31)	27	(24–31)	0.923
SBP, mmHg, median (IQR)	140	(121–160)	148	(130–170)	**0.001**	141	(123–160)	147	(130–168)	**0.002**
DBP, mmHg, median (IQR)	79	(68–90)	80	(70–92)	**0.001**	80	(68–90)	80	(70–92)	0.079
Heart rate, bpm, median (IQR)	81	(69–96)	80	(68–93)	0.111	80	(68–92)	80	(68–93)	0.840
Medical history, n (%)										
Coronary artery disease	495	(40.1)	280	(43.3)	0.185	222	(43.4)	220	(43.0)	0.900
Prior myocardial infarction	295	(23.9)	152	(23.5)	0.842	133	(26.0)	115	(22.5)	0.189
Prior PCI	341	(27.6)	188	(29.1)	0.514	153	(29.9)	149	(29.1)	0.784
Prior CABG	117	(9.5)	76	(11.7)	0.124	55	(10.7)	61	(11.9)	0.554
Prior valvular surgery	53	(4.3)	33	(5.1)	0.427	23	(4.5)	27	(5.3)	0.562
Congestive heart failure	393	(31.8)	231	(35.7)	0.092	172	(33.6)	172	(33.6)	1.000
Decompensated heart failure < 12 months	125	(10.1)	74	(11.4)	0.381	57	(11.1)	53	(10.4)	0.686
Prior ICD	26	(2.1)	13	(2.0)	0.888	12	(2.3)	10	(2.0)	0.666
Prior sICD	4	(0.3)	2	(0.3)	0.956	2	(0.4)	2	(0.4)	1.000
Prior CRT-D	17	(1.4)	10	(1.5)	0.771	7	(1.4)	8	(1.6)	0.795
Prior pacemaker	115	(9.3)	64	(9.9)	0.688	53	(10.4)	49	(9.6)	0.676
Chronic kidney disease	360	(29.2)	228	(35.2)	**0.007**	159	(31.1)	173	(33.8)	0.350
Peripheral artery disease	127	(10.3)	86	(13.3)	0.051	52	(10.2)	70	(13.7)	0.083
Stroke	166	(13.5)	113	(17.5)	**0.020**	80	(15.6)	95	(18.6)	0.213
Liver cirrhosis	22	(1.8)	17	(2.6)	0.222	13	(2.5)	16	(3.1)	0.572
Malignancy	203	(16.5)	80	(12.4)	**0.019**	60	(11.7)	66	(12.9)	0.568
COPD	165	(13.4)	55	(8.5)	**0.002**	46	(9.0)	41	(8.0)	0.575
Cardiovascular risk factors, n (%)										
Arterial hypertension	923	(74.8)	549	(84.9)	**0.001**	416	(81.3)	441	(86.1)	**0.034**
Diabetes mellitus	418	(33.9)	272	(42.0)	**0.001**	184	(35.9)	218	(42.6)	**0.030**
Hyperlipidemia	373	(30.2)	205	(31.7)	0.515	164	(32.0)	166	(32.4)	0.894
Smoking										
Current	250	(20.3)	107	(16.5)	0.051	103	(20.1)	79	(15.4)	**0.050**
Former	245	(19.9)	99	(15.3)	**0.015**	118	(23.0)	78	(15.2)	**0.001**
Family history	131	(10.6)	43	(6.6)	**0.005**	62	(12.1)	32	(6.3)	**0.001**
Comorbidities at index hospitalization, n (%)										
Acute coronary syndrome										
Unstable angina	61	(4.9)	29	(4.5)	0.656	35	(6.8)	24	(4.7)	0.140
STEMI	115	(9.3)	40	(6.2)	**0.019**	59	(11.5)	37	(7.2)	**0.018**
NSTEMI	139	(11.3)	100	(15.5)	**0.010**	63	(12.3)	78	(15.2)	0.174
Acute decompensated heart failure	244	(19.8)	160	(24.7)	**0.013**	99	(19.3)	124	(24.2)	0.058
Cardiogenic shock	26	(2.1)	17	(2.6)	0.473	10	(2.0)	13	(2.5)	0.527
Atrial fibrillation	504	(40.8)	279	(43.1)	0.341	187	(36.5)	213	(41.6)	0.096
Cardiopulmonary resuscitation	27	(2.2)	16	(2.5)	0.694	10	(2.0)	9	(1.8)	0.817
Out-of-hospital	14	(1.1)	6	(0.9)	0.677	6	(1.2)	5	(1.0)	0.762
In-hospital	13	(1.1)	10	(1.5)	0.356	4	(0.8)	4	(0.8)	1.000
Stroke	158	(12.8)	100	(15.5)	0.112	57	(11.1)	77	(15.0)	0.064
Medication on admission, n (%)										
ACE inhibitor	427	(34.6)	240	(37.1)	0.283	188	(36.7)	193	(37.7)	0.746
ARB	258	(20.9)	159	(24.6)	0.069	123	(24.0)	123	(24.0)	1.000
Beta blocker	673	(54.5)	393	(60.7)	**0.010**	295	(57.6)	308	(60.2)	0.409
Aldosterone antagonist	116	(9.4)	62	(9.6)	0.898	47	(9.2)	50	(9.8)	0.749
ARNI	10	(0.8)	5	(0.8)	0.931	5	(1.0)	3	(0.6)	0.478
SGLT2 inhibitor	21	(1.7)	16	(2.5)	0.253	8	(1.6)	12	(2.3)	0.366
Loop diuretics	444	(36.0)	257	(39.7)	0.111	195	(38.1)	197	(38.5)	0.898
Statin	534	(43.3)	317	(49.0)	**0.018**	254	(49.6)	246	(48.0)	0.617
ASA	388	(31.4)	252	(38.9)	**0.001**	175	(34.2)	211	(41.2)	**0.020**
P2Y12 inhibitor	115	(9.3)	59	(9.1)	0.887	57	(11.1)	44	(8.6)	0.173
DOAC	297	(24.1)	146	(22.6)	0.466	125	(24.4)	109	(21.3)	0.234
Vitamin K antagonist	104	(8.4)	58	(9.0)	0.694	38	(7.4)	48	(9.4)	0.260

ACE, angiotensin-converting enzyme; ARB, angiotensin receptor blocker; ARNI, angiotensin receptor–neprilysin inhibitor; ASA, acetylsalicylic acid; CABG, coronary artery bypass grafting; CKD, chronic kidney disease; COPD, chronic obstructive pulmonary disease; CRT-D, cardiac resynchronization therapy with defibrillator; DBP, diastolic blood pressure; DOAC, directly acting oral anticoagulant; IQR, interquartile range; IVSd, interventricular septal end diastole; (N)STEMI, (non-)ST-segment elevation myocardial infarction; SBP, systolic blood pressure; SGLT2, sodium glucose linked transporter 2; (s) ICD, (subcutaneous) implantable cardioverter defibrillator. Level of significance, *p* ≤ 0.05. Bold type indicates statistical significance.

**Table 2:** Heart-failure-related and procedural data.

	Without Propensity Score Matching					With Propensity Score Matching				
	IVSd ≤ 12 mm (*n* = 1234)		IVSd > 12 mm (*n* = 647)		*p* Value	IVSd ≤ 12 mm (*n* = 512)		IVSd > 12 mm (*n* = 512)		*p* Value
Heart failure etiology, n (%)										
Ischemic cardiomyopathy	697	(56.5)	393	(60.7)	**0.001**	314	(61.3)	311	(60.7)	**0.001**
Non-ischemic cardiomyopathy	74	(6.0)	43	(6.6)		32	(6.3)	36	(7.0)	
Hypertensive cardiomyopathy	80	(6.5)	82	(12.7)		35	(6.8)	36	(7.0)	
Congenital heart disease	3	(0.2)	1	(0.2)		3	(0.6)	1	(0.2)	
Valvular heart disease	41	(3.3)	38	(5.9)		15	(2.9)	31	(6.1)	
Tachycardia-associated	79	(6.4)	27	(4.2)		20	(3.9)	18	(3.5)	
Tachymyopathy	22	(1.8)	8	(1.2)		8	(1.6)	7	(1.4)	
Pacemaker-induced cardiomyopathy	12	(1.0)	4	(0.6)		4	(0.8)	3	(0.6)	
Unknown	248	(20.1)	59	(9.1)		89	(17.4)	47	(9.2)	
NYHA functional class, n (%)										
I/II	919	(74.5)	451	(69.7)	0.121	382	(74.6)	360	(70.3)	0.367
III	218	(17.7)	130	(20.1)		88	(17.2)	103	(20.1)	
IV	97	(7.9)	66	(10.2)		42	(8.2)	49	(9.6)	
Echocardiographic data										
LVEF, %, median (IQR)	45 (45–47)		45 (45–47)		0.509	45 (45–47)		45 (45–47)		0.497
Posterior wall, mm, median (IQR)	11 (10–12)		13 (12–14)		**0.001**	11 (10–12)		13 (12–14)		**0.001**
IVSd, mm, median (IQR)	11 (10–12)		14 (13–15)		**0.001**	11 (10–12)		14 (13–15)		**0.001**
LVEDD, mm, median (IQR)	49 (45–54)		48 (44–53)		**0.001**	50 (45–54)		48 (43–52)		**0.001**
TAPSE, mm, median (IQR)	20 (18–23)		20 (17–23)		**0.016**	20 (18–23)		20 (17–23)		**0.032**
LA diameter, mm, median (IQR)	41 (36–46)		44 (39–50)		**0.001**	41 (36–47)		44 (39–50)		**0.001**
LA surface, cm^2^, median (IQR)	21 (17–26)		22 (17–27)		0.082	21 (17–26)		22 (18–27)		0.392
E/A, median (IQR)	0.8 (0.6–1.2)		0.8 (0.6–1.2)		0.213	0.8 (0.6–1.2)		0.8 (0.6–1.2)		0.310
E/E′, median (IQR)	9.0 (6.5–13.0)		10.5 (6.5–15.0)		**0.008**	9.5 (6.5–12.8)		10.0 (6.3–15.0)		0.134
Diastolic dysfunction, n (%)	884	(71.6)	496	(76.7)	**0.019**	391	(76.4)	386	(75.4)	0.715
Moderate–severe aortic stenosis, n (%)	80	(6.5)	100	(15.5)	**0.001**	27	(5.3)	78	(15.2)	**0.001**
Moderate–severe aortic regurgitation, n (%)	39	(3.2)	36	(5.6)	**0.011**	15	(2.9)	30	(5.9)	**0.022**
Moderate–severe mitral regurgitation, n (%)	142	(11.5)	95	(14.7)	**0.049**	60	(11.7)	77	(15.0)	0.119
Moderate–severe tricuspid regurgitation, n (%)	193	(15.6)	109	(16.8)	0.498	73	(14.3)	86	(16.8)	0.262
VCI, mm, median (IQR)	19 (15–25)		20 (16–26)		0.120	19 (13–24)		19 (16–26)		0.149
Aortic root, mm, median (IQR)	32 (29–36)		34 (30–37)		**0.001**	32 (30–35)		34 (30–37)		**0.001**
AV—Vmax, m/s, median, (IQR)	1.46 (1.20–1.80)		1.60 (1.20–2.46)		**0.001**	1.50 (1.20–1.80)		1.60 (1.20–2.46)		**0.009**
Coronary angiography, n (%)	510	(41.3)	275	(42.5)	0.624	235	(45.9)	230	(44.9)	0.754
No evidence of coronary artery disease	107	(21.0)	43	(15.6)	0.228	39	(16.6)	36	(15.7)	0.703
One-vessel disease	98	(19.2)	50	(18.2)		46	(19.6)	40	(17.4)	
Two-vessel disease	99	(19.4)	64	(23.3)		46	(19.6)	55	(23.9)	
Three-vessel disease	206	(40.4)	118	(42.9)		104	(44.3)	99	(43.0)	
CABG	38	(7.5)	29	(10.5)	0.139	21	(8.9)	27	(11.7)	0.321
Chronic total occlusion	59	(11.6)	37	(13.5)	0.442	27	(11.5)	30	(13.0)	0.609
PCI, n (%)	270	(52.9)	157	(57.1)	0.265	136	(57.9)	133	(57.8)	0.992
Sent to CABG, n (%)	29	(5.7)	14	(5.1)	0.727	14	(6.0)	10	(4.3)	0.433
Baseline laboratory values, median (IQR)										
Potassium, mmol/L	3.9 (3.6–4.2)		3.9 (3.6–4.2)		0.427	3.9 (3.6–4.2)		3.9 (3.6–4.2)		0.254
Sodium, mmol/L	139 (137–141)		139 (137–141)		0.393	139 (137–141)		139 (137–141)		0.793
Creatinine, mg/dL	1.05 (0.85–1.42)		1.10 (0.92–1.54)		**0.001**	1.05 (0.86–1.44)		1.10 (0.92–1.49)		**0.016**
eGFR, mL/min/1.73 m^2^	68 (46–89)		62 (43–81)		**0.002**	69 (46–89)		63 (44–80)		**0.011**
Hemoglobin, g/dL	12.5 (10.4–14.0)		12.6 (10.5–14.2)		0.313	12.6 (10.5–14.1)		12.5 (10.6–14.2)		0.803
WBC count, × 10^9^/L	8.28 (6.45)		8.04 (6.42–10.00)		0.417	8.35 (6.56–10.01)		7.97 (6.38–9.78)		0.092
Platelet count, × 10^9^/L	227 (176–287)		223 (178–272)		0.227	224 (179–276)		222 (178–270)		0.372
HbA1c, %	5.9 (5.5–6.8)		6.0 (5.5–6.8)		0.385	5.9 (5.5–6.8)		6.0 (5.5–6.8)		0.677
LDL cholesterol, mg/dL	99 (74–129)		96 (76–120)		0.229	96 (70–130)		96 (74–119)		0.839
HDL cholesterol, md/dL	43 (35–53)		40 (34–50)		**0.010**	42 (34–53)		41 (34–51)		0.296
C-reactive protein, mg/L	13 (3–44)		12 (4–40)		0.911	9 (3–34)		11 (3–38)		0.174
NT-proBNP, pg/mL	2279 (841–5417)		3327 (1141–8436)		**0.005**	2051 (695–5382)		3204 (1030–8796)		**0.005**
NT-proBNP (eGFR corrected), pg/mL	1475 (610–3183)		1797 (707–4096)		0.112	1298 (568–2974)		1805 (679–4210)		**0.038**
Cardiac troponin I, µg/L	0.02 (0.02–0.17)		0.04 (0.02–0.18)		**0.001**	0.3 (0.02–0.29)		0.04 (0.02–0.16)		0.185
Medication at discharge, n (%)										
ACE inhibitor	611	(50.9)	321	(51.3)	0.883	260	(50.8)	263	(51.4)	0.851
ARB	262	(21.8)	163	(26.0)	**0.044**	128	(25.0)	136	(26.6)	0.568
Beta blocker	936	(78.0)	487	(77.8)	0.920	403	(78.7)	395	(77.1)	0.547
Aldosterone antagonist	166	(13.8)	85	(13.6)	0.881	70	(13.7)	72	(14.1)	0.856
ARNI	12	(1.0)	6	(1.0)	0.932	6	(1.2)	5	(1.0)	0.762
SGLT2 inhibitor	39	(3.3)	23	(3.7)	0.635	17	(3.3)	16	(3.1)	0.860
Loop diuretics	446	(46.3)	317	(50.6)	0.080	233	(45.5)	259	(50.6)	0.104
Thiazide diuretics	190	(15.8)	138	(22.0)	**0.001**	89	(17.4)	114	(22.3)	**0.050**
Statin	801	(66.8)	458	(73.2)	**0.005**	368	(71.9)	369	(72.1)	0.945
Digitalis	58	(4.8)	35	(5.6)	0.485	25	(4.9)	25	(4.9)	1.000
Amiodarone	31	(2.6)	19	(3.0)	0.574	12	(2.3)	12	(2.3)	1.000
ASA	593	(49.4)	341	(54.5)	**0.040**	273	(53.3)	281	(54.9)	0.616
P2Y12 inhibitor	376	(31.3)	217	(34.7)	0.149	191	(37.2)	181	(35.4)	0.516
DOAC	392	(32.7)	196	(31.3)	0.556	166	(32.4)	158	(30.9)	0.591
Vitamin K antagonist	83	(6.9)	46	(7.3)	0.733	32	(6.3)	35	(6.8)	0.705

ACE, angiotensin-converting enzyme; ARB, angiotensin receptor blocker; ARNI, angiotensin receptor–neprilysin inhibitor; ASA, acetylsalicylic acid; AV, aortic valve; CABG, coronary artery bypass grafting; DOAC, directly acting oral anticoagulant; eGFR, estimated glomerular filtration rate; HbA1c, glycated hemoglobin; HDL, high-density lipoprotein; IQR, interquartile range; IVSd, interventricular septal end diastole; LA, left atrial; LDL, low-density lipoprotein; LVEDD, left ventricular end-diastolic diameter; LVEF, left ventricular ejection fraction; NT-proBNP, amino-terminal pro-B-type natriuretic peptide; NYHA, New York Heart Association; PCI, percutaneous coronary intervention; TAPSE, tricuspid annular plane systolic excursion; VCI, vena cava inferior; WBC, white blood cell. Level of significance, *p* ≤ 0.05. Bold type indicates statistical significance.

**Table 3:** Correlations of IVSd with laboratory, echocardiographic, and clinical parameters.

	IVSd	
Variables	r	*p* Value
Age	0.102	**0.001**
Body mass index (kg/m^2^)	0.127	**0.001**
LVEDD (mm)	−0.094	**0.001**
Posterior wall (mm)	0.683	**0.001**
TAPSE (mm)	−0.043	0.060
AV—Vmax (m/s)	0.137	**0.001**
NT-proBNP (pg/mL)	0.045	0.268
Creatinine (mg/dL)	0.107	**0.001**
Hemoglobin (g/dL)	0.048	**0.039**

AV, aortic valve; IVSd, interventricular septal end diastole; LVEDD, left ventricular end-diastolic diameter; NT-proBNP, amino-terminal pro-B-type natriuretic peptide; TAPSE, tricuspid annular plane systolic excursion. Level of significance, *p* ≤ 0.05. Bold type indicates statistical significance.

**Table 4:** Multivariate Cox regression analyses with regard to all-cause mortality and heart-failure-related rehospitalization at 30 months.

Variables	All-Cause Mortality			Heart-Failure-Related Rehospitalization		
	HR	95% CI	*p* Value	HR	95% CI	*p* Value
Age > 75 years	2.382	1.894–2.995	**0.001**	1.278	0.943–1.733	0.114
Male	1.333	1.079–1.646	**0.008**	0.835	0.619–1.127	0.239
BMI	0.961	0.940–0.983	**0.001**	1.024	0.996–1.052	0.088
Prior congestive heart failure	1.239	0.964–1.591	0.094	1.383	0.977–1.959	0.068
Decompensated heart failure < 12 months	1.020	0.736–1.413	0.905	1.591	1.080–2.346	**0.019**
Prior acute myocardial infarction	1.118	0.831–1.503	0.462	1.038	0.704–1.532	0.850
Percutaneous coronary intervention	1.111	0.820–1.505	0.498	1.401	0.927–2.118	0.109
COPD	1.184	0.895–1.568	0.237	1.349	0.927–1.962	0.118
Arterial hypertension	0.948	0.718–1.252	0.706	1.254	0.799–1.969	0.326
Diabetes	1.176	0.948–1.459	0.140	1.252	0.925–1.693	0.145
Hemoglobin	0.773	0.737–0.811	**0.001**	0.889	0.833–0.949	**0.001**
NYHA functional class	1.093	0.986–1.211	0.091	1.354	1.174–1.560	**0.001**
Ischemic cardiomyopathy	0.741	0.554–0.992	**0.044**	0.918	0.608–1.385	0.682
Acute myocardial infarction	0.825	0.598–1.138	0.240	0.778	0.503–1.203	0.258
Diastolic dysfunction	0.960	0.766–1.203	0.721	0.770	0.567–1.047	0.095
Beta blockers at discharge	0.706	0.561–0.889	**0.003**	1.225	0.839–1.790	0.294
SGLT2 inhibitors at discharge	1.104	0.538–2.264	0.788	0.927	0.400–2.150	0.861
ACE inhibitor/ARB/ARNI at discharge	0.661	0.531–0.823	**0.001**	0.963	0.682–1.360	0.831
LVEDD	0.998	0.993–1.002	0.344	1.002	0.999–1.005	0.162
IVSd ≤ 12 mm vs. >12 mm	1.001	0.985–1.017	0.930	1.340	1.002–1.792	**0.049**

ACE, angiotensin-converting enzyme; ARB, angiotensin receptor blocker; ARNI, angiotensin receptor–neprilysin inhibitor; BMI, body mass index; CI, confidence interval; COPD, chronic obstructive pulmonary disease; HR, hazard ratio; IVSd, interventricular septal end diastole; LVEDD, left ventricular end-diastolic diameter; NYHA, New York Heart Association; SGLT, sodium glucose linked transporter. Level of significance, *p* ≤ 0.05. Bold type indicates statistical significance.

**Table 5:** Multivariate Cox regression analyses of different IVSd categories with regard to all-cause mortality and heart-failure-related rehospitalization at 30 months.

Variables	All-Cause Mortality			Heart-Failure-Related Rehospitalization		
	HR	95% CI	*p* Value	HR	95% CI	*p* Value
Age > 75 years	2.375	1.889–2.987	**0.001**	1.265	0.933–1.715	0.130
Males	1.319	1.065–1.633	**0.011**	0.818	0.606–1.105	0.190
BMI	0.961	0.939–0.983	**0.001**	1.023	0.996–1.052	0.098
Prior congestive heart failure	1.239	0.964–1.591	0.094	1.400	0.989–1.983	0.058
Decompensated heart failure < 12 months	1.017	0.734–1.410	0.918	1.601	1.087–2.359	**0.017**
Prior acute myocardial infarction	1.122	0.835–1.509	0.446	1.032	0.700–1.521	0.874
Percutaneous coronary intervention	1.111	0.820–1.505	0.499	1.394	0.924–2.104	0.113
COPD	1.197	0.903–1.588	0.211	1.343	0.924–1.953	0.122
Arterial hypertension	0.934	0.706–1.236	0.632	1.232	0.785–1.933	0.364
Diabetes	1.175	0.946–1.459	0.145	1.236	0.914–1.671	0.168
Hemoglobin	0.772	0.736–0.810	**0.001**	0.889	0.832–0.949	**0.001**
NYHA functional class	1.090	0.983–1.208	0.102	1.351	1.173–1.557	**0.001**
Ischemic cardiomyopathy	0.744	0.556–0.994	**0.046**	0.926	0.612–1.401	0.717
Acute myocardial infarction (at index)	0.821	0.595–1.132	0.229	0.779	0.503–1.205	0.262
Diastolic dysfunction	0.963	0.768–1.208	0.746	0.772	0.568–1.050	0.099
Beta blockers at discharge	0.707	0.561–0.891	**0.003**	1.231	0.843–1.799	0.282
SGLT2 inhibitors at discharge	1.094	0.534–2.244	0.806	0.925	0.399–2.143	0.855
ACE inhibitors/ARB/ARNI at discharge	0.663	0.532–0.826	**0.001**	0.961	0.680–1.358	0.821
LVEDD	0.998	0.993–1.002	0.364	1.002	0.999–1.005	0.139
IVSd < 10 mm	(reference group)		0.854	(reference group)		0.081
IVSd 10–12 mm	1.029	0.734–1.441	0.870	1.463	0.832–2.571	0.186
IVSd > 12–14 mm	1.061	0.734–1.534	0.753	1.743	0.959–3.167	0.068
IVSd > 14 mm	1.187	0.762–1.850	0.448	2.261	1.153–4.433	**0.018**

ACE, angiotensin-converting enzyme; ARB, angiotensin receptor blocker; ARNI, angiotensin receptor–neprilysin inhibitor; BMI, body mass index; CI, confidence interval; COPD, chronic obstructive pulmonary disease; HR, hazard ratio; IVSd, interventricular septal end diastole; LVEDD, left ventricular end-diastolic diameter; NYHA, New York Heart Association; SGLT, sodium glucose linked transporter. Level of significance, *p* ≤ 0.05. Bold type indicates statistical significance.

## Data Availability

The datasets used and/or analyzed in the current study are available from the corresponding author upon reasonable request.

## Appendix A

**Table A1:** Follow-up data and primary and secondary endpoints.

	Without Propensity Score Matching						
	IVSd ≤ 12 mm (*n* = 1234)		IVSd > 12 mm (*n* = 647)		HR	95% CI	*p* Value
Primary endpoint, n (%)							
- All-cause mortality at 30 months	360	(29.2)	204	(31.5)	1.067	0.898–1.267	0.460
Secondary endpoints, n (%)							
- All-cause mortality, in-hospital	34	(2.8)	21	(3.2)	1.409	0.812–2.444	0.223
- All-cause mortality at 12 months	249	(20.2)	129	(19.9)	0.984	0.795–1.217	0.880
- Heart-failure-related rehospitalization at 30 months	145	(12.1)	97	(15.5)	1.303	1.008–1.685	**0.044**
- Cardiac rehospitalization at 30 months	253	(21.1)	148	(23.6)	1.139	0.930–1.395	0.209
- Revascularization at 30 months	83	(6.9)	41	(6.5)	0.945	0.650–1.373	0.766
- Acute myocardial infarction at 30 months	30	(2.5)	23	(3.7)	1.458	0.847–2.511	0.173
- Stroke at 30 months	32	(2.7)	17	(2.7)	1.009	0.560–1.817	0.976
- MACCEs at 30 months	447	(36.2)	255	(39.4)	1.081	0.927–1.261	0.320
Follow-up data, median (IQR)							
- Hospitalization time, days	9 (5–15)		9 (5–15)		-	-	0.952
- ICU time, days	0 (0–1)		0 (0–1)		-	-	0.249
- Follow-up time, days	948 (416–1647)		955 (449–1738)		-	-	0.256
	**With Propensity Score Matching**						
	**IVSd ≤ 12 mm** **(*n* = 512)**		**IVSd > 12 mm** **(*n* = 512)**		**HR**	**95% CI**	***p* Value**
Primary endpoint, n (%)							
- All-cause mortality at 30 months	126	(24.6)	151	(29.5)	1.205	0.951–1.526	0.122
Secondary endpoints, n (%)							
- All-cause mortality, in-hospital	0	(0.0)	0	(0.0)	-	-	-
- All-cause mortality at 12 months	81	(15.8)	91	(17.8)	1.139	0.844–1.536	0.396
- Heart-failure-related rehospitalization at 30 months	60	(11.7)	82	(16.0)	1.399	1.002–1.951	**0.048**
- Cardiac rehospitalization at 30 months	114	(22.3)	120	(23.4)	1.059	0.819–1.368	0.663
- Revascularization at 30 months	41	(8.0)	33	(6.4)	0.795	0.503–1.258	0.328
- Acute myocardial infarction at 30 months	16	(3.1)	18	(3.5)	1.107	0.565–2.172	0.766
- Stroke at 30 months	10	(2.0)	12	(2.3)	1.189	0.514–2.753	0.686
- MACCEs at 30 months	165	(32.2)	192	(37.5)	1.176	0.955–1.448	0.127
Follow-up data, median (IQR)							
- Hospitalization time, days	8 (5–14)		8 (5–15)		-	-	0.333
- ICU time, days	0 (0–1)		0 (0–1)		-	-	0.580
- Follow-up time, days	1045 (512–1775)		991 (509–1756)		-	-	0.892

CI, confidence interval; HR, hazard ratio; ICU, intensive care unit; IVSd, interventricular septal end diastole; MACCEs, major adverse cardiac and cerebrovascular events. Level of significance, *p* ≤ 0.05. Bold type indicates statistical significance.

## References

[1] Groenewegen A., Rutten F.H., Mosterd A., Hoes A.W. (2020). Epidemiology of heart failure. Eur. J. Heart Fail..

[2] Savarese G., Becher P.M., Lund L.H., Seferovic P., Rosano G.M.C., Coats A.J.S. (2023). Global burden of heart failure: A comprehensive and updated review of epidemiology. Cardiovasc. Res..

[3] Emmons-Bell S., Johnson C., Roth G. (2022). Prevalence, incidence and survival of heart failure: A systematic review. Heart.

[4] Roger V.L. (2013). Epidemiology of heart failure. Circ. Res..

[5] Kemp C.D., Conte J.V. (2012). The pathophysiology of heart failure. Cardiovasc. Pathol..

[6] Tanai E., Frantz S. (2016). Pathophysiology of Heart Failure. Compr. Physiol..

[7] Tan L.B., Hall A.S. (1994). Cardiac remodelling. Br. Heart J..

[8] Kurrelmeyer K., Kalra D., Bozkurt B., Wang F., Dibbs Z., Seta Y., Baumgarten G., Engle D., Sivasubramanian N., Mann D.L. (1998). Cardiac remodeling as a consequence and cause of progressive heart failure. Clin. Cardiol..

[9] Spinale F.G., Janicki J.S., Zile M.R. (2013). Membrane-associated matrix proteolysis and heart failure. Circ. Res..

[10] Dadson K., Kovacevic V., Rengasamy P., Kim G.H., Boo S., Li R.K., George I., Schulze P.C., Hinz B., Sweeney G. (2016). Cellular, structural and functional cardiac remodelling following pressure overload and unloading. Int. J. Cardiol..

[11] Cohn J.N., Ferrari R., Sharpe N. (2000). Cardiac remodeling—Concepts and clinical implications: A consensus paper from an international forum on cardiac remodeling. Behalf of an International Forum on Cardiac Remodeling. J. Am. Coll. Cardiol..

[12] Triposkiadis F., Xanthopoulos A., Boudoulas K.D., Giamouzis G., Boudoulas H., Skoularigis J. (2022). The Interventricular Septum: Structure, Function, Dysfunction, and Diseases. J. Clin. Med..

[13] Kaul S. (1986). The interventricular septum in health and disease. Am. Heart J..

[14] Buckberg G., Hoffman J.I.E. (2014). Right ventricular architecture responsible for mechanical performance: Unifying role of ventricular septum. J. Thorac. Cardiovasc. Surg..

[15] Loncaric F., Nunno L., Mimbrero M., Marciniak M., Fernandes J.F., Tirapu L., Fabijanovic D., Sanchis L., Doltra A., Cikes M. (2020). Basal Ventricular Septal Hypertrophy in Systemic Hypertension. Am. J. Cardiol..

[16] Kutyifa V., Solomon S.D., Bourgoun M., Shah A.M., Pouleur A.-C., Knappe D., McNitt S., Wang P.J., Merkely B., Pfeffer M. (2013). Effects of cardiac resynchronization therapy on left ventricular mass and wall thickness in mild heart failure patients in MADIT-CRT. Heart Rhythm..

[17] Hill L., Monaghan M., Richardson P. (1979). Regression of left ventricular hypertrophy during treatment with antihypertensive agents. Br. J. Clin. Pharmacol..

[18] Huang B.T., Peng Y., Liu W., Zhang C., Huang F.Y., Wang P.J., Zuo Z.L., Liao Y.B., Chai H., Huang K.S. (2015). Increased interventricular septum wall thickness predicts all-cause death in patients with coronary artery disease. Intern. Med. J..

[19] McDonagh T.A., Metra M., Adamo M., Gardner R.S., Baumbach A., Bohm M., Burri H., Butler J., Celutkiene J., Chioncel O. (2021). 2021 ESC Guidelines for the diagnosis and treatment of acute and chronic heart failure. Eur. Heart J..

[20] McMurray J.J., Packer M., Desai A.S., Gong J., Lefkowitz M.P., Rizkala A.R., Rouleau J.L., Shi V.C., Solomon S.D., Swedberg K. (2014). Angiotensin-neprilysin inhibition versus enalapril in heart failure. N. Engl. J. Med..

[21] Consensus Trial Study Group (1987). Effects of enalapril on mortality in severe congestive heart failure. Results of the Cooperative North Scandinavian Enalapril Survival Study (CONSENSUS). N. Engl. J. Med..

[22] Hjalmarson A., Goldstein S., Fagerberg B., Wedel H., Waagstein F., Kjekshus J., Wikstrand J., El Allaf D., Vitovec J., Aldershvile J. (2000). Effects of controlled-release metoprolol on total mortality, hospitalizations, and well-being in patients with heart failure: The Metoprolol CR/XL Randomized Intervention Trial in congestive heart failure (MERIT-HF). MERIT-HF Study Group. JAMA.

[23] Cohn J.N., Tognoni G., Valsartan Heart Failure Trial I. (2001). A randomized trial of the angiotensin-receptor blocker valsartan in chronic heart failure. N. Engl. J. Med..

[24] Mondillo S., Galderisi M., Mele D., Cameli M., Lomoriello V.S., Zaca V., Ballo P., D’Andrea A., Muraru D., Losi M. (2011). Speckle-tracking echocardiography: A new technique for assessing myocardial function. J. Ultrasound Med..

[25] Cameli M., Mondillo S., Solari M., Righini F.M., Andrei V., Contaldi C., De Marco E., Di Mauro M., Esposito R., Gallina S. (2016). Echocardiographic assessment of left ventricular systolic function: From ejection fraction to torsion. Heart Fail. Rev..

[26] Onishi T., Saha S.K., Delgado-Montero A., Ludwig D.R., Onishi T., Schelbert E.B., Schwartzman D., Gorcsan J. (2015). Global longitudinal strain and global circumferential strain by speckle-tracking echocardiography and feature-tracking cardiac magnetic resonance imaging: Comparison with left ventricular ejection fraction. J. Am. Soc. Echocardiogr..

[27] Chen J.S., Pei Y., Li C.E., Li N.Y., Guo T., Yu J. (2020). Prognostic value of heart failure echocardiography index in HF patients with preserved, mid-ranged and reduced ejection fraction. BMC Cardiovasc. Disord..

[28] Yamaguchi S., Shimabukuro M., Abe M., Arakaki T., Arasaki O., Ueda S. (2019). Comparison of the prognostic values of three calculation methods for echocardiographic relative wall thickness in acute decompensated heart failure. Cardiovasc. Ultrasound.

[29] Schmitt A., Schupp T., Reinhardt M., Abel N., Lau F., Forner J., Ayoub M., Mashayekhi K., Weiß C., Akin I. (2023). Prognostic impact of acute decompensated heart failure in patients with heart failure and mildly reduced ejection fraction. Eur. Heart J. Acute Cardiovasc. Care.

[30] Popescu B.A., Andrade M.J., Badano L.P., Fox K.F., Flachskampf F.A., Lancellotti P., Varga A., Sicari R., Evangelista A., Nihoyannopoulos P. (2009). European Association of Echocardiography recommendations for training, competence, and quality improvement in echocardiography. Eur. J. Echocardiogr..

[31] Lancellotti P., Tribouilloy C., Hagendorff A., Popescu B.A., Edvardsen T., Pierard L.A., Badano L., Zamorano J.L. (2013). Recommendations for the echocardiographic assessment of native valvular regurgitation: An executive summary from the European Association of Cardiovascular Imaging. Eur. Heart J. Cardiovasc. Imaging.

[32] Lang R.M., Badano L.P., Mor-Avi V., Afilalo J., Armstrong A., Ernande L., Flachskampf F.A., Foster E., Goldstein S.A., Kuznetsova T. (2015). Recommendations for cardiac chamber quantification by echocardiography in adults: An update from the American Society of Echocardiography and the European Association of Cardiovascular Imaging. Eur. Heart J. Cardiovasc. Imaging.

[33] Stewart M.H., Lavie C.J., Shah S., Englert J., Gilliland Y., Qamruddin S., Dinshaw H., Cash M., Ventura H., Milani R. (2018). Prognostic Implications of Left Ventricular Hypertrophy. Prog. Cardiovasc. Dis..

[34] Eliakim-Raz N., Prokupetz A., Gordon B., Shochat T., Grossman A. (2016). Interventricular Septum and Posterior Wall Thickness Are Associated With Higher Systolic Blood Pressure. J. Clin. Hypertens..

[35] Nakamura M., Sadoshima J. (2018). Mechanisms of physiological and pathological cardiac hypertrophy. Nat. Rev. Cardiol..

[36] Weber K.T., Janicki J.S., Shroff S.G., Pick R., Chen R.M., Bashey R.I. (1988). Collagen remodeling of the pressure-overloaded, hypertrophied nonhuman primate myocardium. Circ. Res..

[37] Iyer N.R., Le T.T., Kui M.S.L., Tang H.C., Chin C.T., Phua S.K., Bryant J.A., Pua C.J., Ang B., Toh D.F. (2022). Markers of Focal and Diffuse Nonischemic Myocardial Fibrosis Are Associated with Adverse Cardiac Remodeling and Prognosis in Patients With Hypertension: The REMODEL Study. Hypertension.

[38] Takano H., Hasegawa H., Nagai T., Komuro I. (2003). Implication of cardiac remodeling in heart failure: Mechanisms and therapeutic strategies. Intern. Med..

[39] Gardin J.M., McClelland R., Kitzman D., Lima J.A., Bommer W., Klopfenstein H.S., Wong N.D., Smith V.E., Gottdiener J. (2001). M-mode echocardiographic predictors of six- to seven-year incidence of coronary heart disease, stroke, congestive heart failure, and mortality in an elderly cohort (the Cardiovascular Health Study). Am. J. Cardiol..

[40] Quinones M.A., Greenberg B.H., Kopelen H.A., Koilpillai C., Limacher M.C., Shindler D.M., Shelton B.J., Weiner D.H. (2000). Echocardiographic predictors of clinical outcome in patients with left ventricular dysfunction enrolled in the SOLVD registry and trials: Significance of left ventricular hypertrophy. Studies of Left Ventricular Dysfunction. J. Am. Coll. Cardiol..

[41] Hawkins N.M., Wang D., McMurray J.J., Pfeffer M.A., Swedberg K., Granger C.B., Yusuf S., Pocock S.J., Ostergren J., Michelson E.L. (2007). Prevalence and prognostic implications of electrocardiographic left ventricular hypertrophy in heart failure: Evidence from the CHARM programme. Heart.

[42] Shah A.M., Cikes M., Prasad N., Li G., Getchevski S., Claggett B., Rizkala A., Lukashevich I., O’Meara E., Ryan J.J. (2019). Echocardiographic Features of Patients With Heart Failure and Preserved Left Ventricular Ejection Fraction. J. Am. Coll. Cardiol..

[43] Apostolakis S., Sullivan R.M., Olshansky B., Lip G.Y. (2014). Left ventricular geometry and outcomes in patients with atrial fibrillation: The AFFIRM Trial. Int. J. Cardiol..

